# A Multiplex Real-Time PCR Assay to Diagnose and Separate *Helicoverpa armigera* and *H*. *zea* (Lepidoptera: Noctuidae) in the New World

**DOI:** 10.1371/journal.pone.0142912

**Published:** 2015-11-11

**Authors:** Todd M. Gilligan, Luke R. Tembrock, Roxanne E. Farris, Norman B. Barr, Marja J. van der Straten, Bart T. L. H. van de Vossenberg, Eveline Metz-Verschure

**Affiliations:** 1 USDA-APHIS-PPQ-Science & Technology, Identification Technology Program, Fort Collins, Colorado, United States of America; 2 Department of Biology, Colorado State University, Fort Collins, Colorado, United States of America; 3 USDA-APHIS-PPQ-Science & Technology, Mission Laboratory, Edinburg, Texas, United States of America; 4 National Plant Protection Organization, Netherlands Food and Consumers Product Safety Authority, Ministry of Economic Affairs, Wageningen, The Netherlands; Natural Resources Canada, CANADA

## Abstract

The Old World bollworm, *Helicoverpa armigera* (Hübner), and the corn earworm, *H*. *zea* (Boddie), are two of the most important agricultural pests in the world. Diagnosing these two species is difficult—adults can only be separated with a complex dissection, and larvae cannot be identified to species using morphology, necessitating the use of geographic origin for identification in most instances. With the discovery of *H*. *armigera* in the New World, identification of immature *Helicoverpa* based on origin is no longer possible because *H*. *zea* also occurs in all of the geographic regions where *H*. *armigera* has been discovered. DNA barcoding and restriction fragment length polymorphism (RFLP) analyses have been reported in publications to distinguish these species, but these methods both require post-PCR processing (i.e., DNA sequencing or restriction digestion) to complete. We report the first real-time PCR assay to distinguish these pests based on two hydrolysis probes that bind to a segment of the internal transcribed spacer region 2 (ITS2) amplified using a single primer pair. One probe targets *H*. *armigera*, the second probe targets *H*. *zea*, and a third probe that targets a conserved segment of 18S rDNA is used as a control of DNA quality. The assay can be completed in 50 minutes when using isolated DNA and is successfully tested on larvae intercepted at ports of entry and adults captured during domestic surveys. We demonstrate that the assay can be run in triplex with no negative effects on sensitivity, can be run using alternative real-time PCR reagents and instruments, and does not cross react with other New World Heliothinae.

## Introduction

The Old World bollworm, *Helicoverpa armigera* (Hübner), and the corn earworm, *H*. *zea* (Boddie), are two of the most important agricultural pests in the world. A native of the Old World, *H*. *armigera* is the widest distributed species in the genus *Helicoverpa* [1–2], occurring from the Canary Islands east across much of Europe, Africa, Asia, and Australasia to the islands of Tonga in the southern Pacific Ocean. In the New World, *H*. *zea* is distributed across much of North and South America and the Caribbean [1]. Both species are highly polyphagous, with *H*. *armigera* feeding on hosts in 68 plant families and *H*. *zea* feeding on hosts in 36 plant families [3]. Preferred hosts for both species include important agricultural crops, such as corn, cotton, soybean, tobacco, tomato, and many others [3–4].

In late 2012/early 2013, an outbreak of *Helicoverpa* larvae was observed damaging soybean and cotton in the Cerrado region of central Brazil [4–6]. Adults reared from larvae were identified using morphology [5], and later DNA sequence data [6–7], as *H*. *armigera*. Subsequent reports confirmed *H*. *armigera* as present throughout much of Brazil [4, 7–9], and this species was also reported from Argentina, Bolivia, Paraguay, and Uruguay [10–11]. In September, 2014, authors of this study from the United States Department of Agriculture (USDA) Mission Lab confirmed the first U.S. detection of *H*. *armigera* in San Germán, Puerto Rico using DNA sequence data [12]. Between April, 2014 and February, 2015 authors of this study from the National Plant Protection Organization (NPPO) of the Netherlands confirmed interceptions of *H*. *armigera* from the Dominican Republic and Peru using DNA sequence data. Netherlands NPPO also identified two larvae intercepted from Surinam in 2011 and 2012 as *H*. *armigera* using sequence data; however, at that time presence of *H*. *armigera* in the New World was not known, and therefore the reported origin of these consignments was assumed to be incorrect. These findings corroborate analysis of *H*. *armigera* mitochondrial DNA (mtDNA) sequence data from Brazil that implies multiple invasion events or invasion several years prior to first discovery [9]. In June and July, 2015, three individuals of *H*. *armigera* were discovered in Florida; these are the first reports of *H*. *armigera* from the Continental U.S. [13–14].

Adults of both *H*. *armigera* and *H*. *zea* are morphologically variable and cannot be identified reliably without genitalic dissection [15]. Brambila [16] provides step-by-step instructions for dissecting and diagnosing *H*. *armigera* and *H*. *zea* using male genitalia. This process is very tedious and time consuming, even for a lepidopteran specialist, especially when dealing with potentially hundreds of moths per trap. Identification of *Helicoverpa* larvae is much more problematic than the identification of adults. Gilligan and Passoa [17] compared the head chaetotaxy, mandibles, hypopharyngeal complex, body coloration and markings, body chaetotaxy, pinacula size and shape, setal color, cuticle texture, and crochet counts and arrangement for various instars of *H*. *armigera* and *H*. *zea* and could not identify any morphological characters that would reliably separate larvae of these two species.

For efficient diagnosis of adults captured during domestic surveys and larvae (and other immature stages) intercepted at ports of entry, means of identification other than morphology are necessary. Molecular techniques have been used to diagnosis these two pests based on variation in the mitochondrial genome. For example, Mastrangelo et al. [4] used DNA barcoding to distinguish between *H*. *armigera* and *H*. *zea* in Brazil. Behere et al. [18] developed a restriction fragment length polymorphism (RFLP) assay using two regions of mitochondrial DNA to distinguish between *H*. *armigera*, *H*. *assulta*, *H*. *punctigera*, as well as *H*. *zea*. These techniques, however, require post-PCR analysis steps that increase the processing time for samples. Restriction digestion and sequencing of PCR products add several hours or days to the time required to complete an analysis.

Real-time PCR [19–20] is a molecular method that can be used for the detection and diagnosis of biological organisms. The benefits of using real-time PCR versus conventional PCR include: reduced assay time; elimination of post-PCR electrophoresis; potential of scaling for high throughput testing; and increased sensitivity and specificity when using a quenched dye system (such as dual-labeled hydrolysis probes) [21]. In addition, real-time PCR eliminates the need to process and sequence the final PCR product, the lengthiest step in DNA barcoding. We have previously developed a real-time PCR assay to diagnose economically important Tortricidae using the internal transcribed spacer region 2 (ITS2) locus as a diagnostic marker and the 18S rDNA locus as an internal control [22]. Here we apply a similar method to develop a real-time PCR assay for diagnosing and separating *H*. *armigera* and *H*. *zea* in the New World.

## Materials and Methods

### Heliothinae collection and identification

Specimens used in this study are summarized in Table 1. A total of 452 Heliothinae representing 18 species were used to develop the real-time PCR assay. One hundred and thirty-nine *H*. *armigera* adults and larvae were obtained from: port interceptions in the Netherlands and U.S.; fresh collections in South Africa; and colleagues in Australia, Brazil, Spain, and South Africa. Two hundred and fifty-eight *H*. *zea* adults and larvae were obtained from: port interceptions in the U.S.; fresh collections in various locations in the U.S.; a lab colony at Mississippi State University; and various USDA Cooperative Agricultural Survey (CAPS) programs, primarily those in Colorado, Florida, and Mississippi. Fifty-five other Heliothinae were obtained from: port interceptions in the U.S.; fresh collections in various locations in the U.S.; colleagues in Spain and South Africa; the C. P. Gillette Museum of Arthropod Diversity at Colorado State University; and the Smithsonian Institution. Field collections in the U.S. were on private land (with permission of the land owner) or public land not requiring a collecting permit (National Forest, National Grassland, or BLM public land). Field collections in Gauteng Province, South Africa did not require a collecting permit under the Nature Conservation Ordinance of 1983 or the South African National Environmental Management: Biodiversity Act of 2004. Specimens provided by colleagues from other foreign countries were collected on private land where no collecting permit was required, or were sourced from experimental colonies. Specimens from port interceptions and USDA CAPS surveys were obtained under the authority of the USDA and Netherlands NPPO. No endangered or protected species were collected for this study. The intercepted and CAPS specimens are representative of insect material expected from sampling at ports of entry and during surveys. The majority of larvae and fresh collected specimens were stored in >95% alcohol in microcentrifuge tubes, pinned adult specimens were stored dry, and adults from CAPS traps were stored dry in plastic bags at −50°C.

**Table 1:** Taxa sampled for study with country of origin, life stage, and number of specimens.

Taxon	Country	Life Stage	Quantity
*Helicoverpa armigera* (Hübner, 1805)	Australia	adults, larvae	96
	Brazil	larvae	5
	Ethiopia	larva	1
	India	larvae	2
	Israel	adults, larvae	13
	Italy	larvae	2
	Jordan	larva	1
	Kenya	adults, larvae	11
	Macedonia	larva	1
	Netherlands	larvae	16
	Pakistan	larva	1
	Palestinian Territory	larvae	2
	South Africa	adults	29
	Spain	adults	10
	Thailand	larvae	2
	Uganda	adult, larvae	3
	Zimbabwe	larvae	7
*Helicoverpa zea* (Boddie, 1850)	Brazil	larvae	5
	Dominican Republic	larvae	6
	Guatemala	larva	1
	Mexico	larvae	5
	Trinidad and Tobago	larvae	2
	U.S.A.	adults, larvae	297
*Chloridea subflexa* (Guenée, 1852)	Mexico	adult	1
	U.S.A.	adults	4
*Chloridea virescens* (Fabricius, 1777)	U.S.A.	adults	5
*Helicoverpa hawaiiensis* (Quaintance & Brues, 1905)	U.S.A.	adults	5
*Helicoverpa titicacae* Hardwick, 1965	Bolivia	adults	4
*Helicoverpa* sp. #1	Bolivia	adult	1
*Helicoverpa* sp. #2	South Africa	adult	1
*Heliocheilus paradoxus* Grote, 1865	U.S.A.	adults	8
*Heliothis peltigera* ([Denis & Schiffermüller], 1775)	Spain	adult	1
*Heliothis phloxiphaga* Grote & Robinson, 1867	U.S.A.	adults	10
*Heliothis* sp. #1	South Africa	adults	3
*Schinia chryselloides* Pogue & Harp, 2005	U.S.A.	adult	1
*Schinia errans* Smith, 1883	U.S.A.	adult	1
*Schinia grandimedia* Hardwick, 1996	U.S.A.	adult	1
*Schinia hulstia* Tepper, 1883	U.S.A.	adult	1
*Schinia mortua* (Grote, 1865)	U.S.A.	adult	1
*Schinia reniformis* Smith, 1900	U.S.A.	adult	1

Identification of specimens was performed using genitalic dissection (adults only) [15] and/or sequencing of cytochrome c oxidase1 (COI) DNA barcodes (adults and larvae) [23]. Amplifications of COI were performed using the primers LepF1/LepR1 [24]. PCR conditions and sequencing steps were identical to those described below for 18s rDNA and ITS2. Edited DNA barcodes were identified using the “BOLD Identification System” of www.boldsystems.org [25]. In all cases, the DNA barcode identifications agreed with the morphological identification and/or real-time PCR ITS2 diagnosis.

All DNA sequences generated for this study were submitted to GenBank under accession numbers KT945996–KT946005 (18S), KT946006–KT946021 (ITS2), and KT946022–KT946127 (COI).

### DNA extraction, conventional PCR, and sequencing

Total genomic DNA was extracted using a Qiagen DNeasy Blood and Tissue Kit (Qiagen, Valencia, Calif.). Tissue used for the extraction varied by specimen type: one to three legs from dried pinned adults; a portion of the thorax for adults in alcohol; two to three abdominal segments for late instar larvae; and the entire larva for early instar larvae. For single column extractions, tissue samples were crushed dry in a 1.5 ml microcentrifuge tube, incubated in a solution of 180 μl Buffer ATL and 20 μl Proteinase K overnight at 56°C (in a dry bath), and eluted in 100 μl of AE buffer after following the manufacturer’s recommended protocol. For plate extractions, tissue samples were chopped and placed in 1.2 ml tubes in deep 96-well plates and incubated overnight at 80°C (56°C dry bath equivalent) in an Eppendorf ThermoMixer (Eppendorf AG, Hamburg, Germany). The rest of the extraction followed the manufacturer’s recommended protocol with 96-well filter plates (Epoch Life Science, Missouri City, Texas) substituted for individual columns. To avoid possible contamination, all equipment and materials were sanitized between specimens and filter tips were used to handle any liquids containing DNA. No-tissue extraction controls were used for each extraction batch/plate when possible. DNA concentration and absorbance for a representative number of samples was estimated with a NanoDrop 1000 Ver. 3.7.0 spectrophotometer (Thermo Scientific/NanoDrop Wilmington, Delaware). Two readings were taken for each sample.

Conventional PCR used a Biometra T3000 (Biometra GmbH, Goettingen, Germany) or Bio-Rad C1000 Touch (Bio-Rad Laboratories, Inc., Hercules, Calif.) thermal cycler. PCR reactions were performed with TaKaRa Ex Taq HS polymerase (Takara Bio, Shiga, Japan) in total volumes of 50 μl using the manufacturer’s recommended volumes of 10X Ex Taq buffer, dNTP mixture, and water. PCR conditions included an initial denaturation step of 94°C (3 min), 32 cycles of 94°C (20 sec) / 50°C (20 sec) / 72°C (30 sec), and an extension step of 72°C (5 min). PCR products were purified with a Qiagen QIAquick PCR Purification Kit (Qiagen, Valencia, Calif.). Purified PCR products were sequenced by the University of Chicago Cancer Research Center DNA Sequencing Facility using an Applied Biosystems 3730XL DNA sequencer (Applied Biosystems, Foster City, Calif.). The same primers used for PCR were also used for sequencing. Individual contigs were assembled, trimmed, and aligned using Geneious Pro 6.1.6 (Biomatters, Auckland, New Zealand) [26]. Following the methods described in Barr et al. [27], select ITS2 fragments were amplified, cloned, and purified prior to sequencing to generate multiple copies from a single moth.

### Primer and probe design

Following Barr et al. [22, 27], the ITS2 locus was selected as a potential diagnostic marker and the 18S rDNA locus was selected as a control. Conventional PCR was used to test the Barr et al. [22] 18S rDNA real-time PCR primers on four samples of *H*. *armigera*, four samples of *H*. *zea*, and two *Heliothis* samples. Seven samples of *H*. *armigera*, five samples of *H*. *zea*, and two *Heliothis* samples were used to sequence a region of 5.8S-ITS2-28S using the ITSF/ITSR primers [22, 27], and sequences generated using these primers were loaded into Geneious. Primer3 [28] was used to design internal primers to amplify a smaller region of the ITS2 locus that would maximize differences between *H*. *armigera* and *H*. *zea*. Primers were designed to avoid regions of intragenomic variation detected in ITS2 for several individuals. After testing several primer combinations, the region amplified by primers 425F/568R (this study) was selected as appropriate for use in real-time PCR. The 425F/568R primers were tested with conventional PCR and sequenced for an additional 19 individuals of *H*. *armigera*, 11 individuals of *H*. *zea*, and three *Heliothis* species (four individuals total).

Geneious was used to manually locate regions in the ITS2 sequence data suitable for placement of species-specific hydrolysis probes. Because *H*. *armigera* and *H*. *zea* are closely related [15], few regions of interspecific variability were found, and initial probes were designed with locked nucleic acids (LNAs) in order to increase specificity and melting temperatures [29–30]. Later probes were designed without LNAs to decrease cost and increase vendor selection for ordering probes. Separate probes for *H*. *armigera* and *H*. *zea* with different fluorophores were designed that targeted two different sequences of ITS2 within the 425F/568R region.

All primers and probes used in this study are listed in Table 2. Melting temperatures (Tm; salt adjusted) were calculated with OligoCalc [31]. Primers were ordered from Integrated DNA Technologies (IDT; Coralville, Iowa) and stored in 1 × TE buffer or sterile water at −50°C; working stocks were diluted to 10 μM in sterile water and stored at −20°C. The 18S rDNA and ITS2 primers generate amplicons of 68 and 140–143 bp, respectively. LNA probes were ordered from IDT and non-LNA probes were ordered from Biosearch Technologies (Petaluma, Calif.) Probes were diluted to 8 μM in sterile water and stored at −20°C. The ITS2 *H*. *armigera* probe was labeled with FAM and the *H*. *zea* probe was labeled with HEX (both with BHQ-1 as quencher). The 18S rDNA control probe was designed identical to that of Barr et al. [22] with the exception of Quasar 670 (= Cy5; BHQ-2 as quencher) replacing CAL Red 610 for greater compatibility with real-time PCR machines that use the Red 610 channel for calibration. All probes were HPLC purified.

**Table 2:** Primers and probes used in this study (Tm = melting temperature in °C).

Name	Description	Sequence	Tm (°C)	Source
RT-18S-F2	18S forward primer for real-time PCR	5'-ACCGCCCTAGTTCTAACCGTAAA	62.9	Barr et al. [22, 27]
RT-18S-R2	18S reverse primer for real-time PCR	5'-CCGCCGAGCCATTGTAGTAA	60.5	Barr et al. [22]
RT-18S-P2	18S real-time PCR probe	5'-Quasar 670-TGTCATCTAGCGATCCGCCGA-BHQ-2	63.2	Barr et al. [22]/This study
ITSF	5.8S-ITS2-28S	5'-TTGAACATCGACATTTCGAACGCAC	64.1	Barr et al. [22, 27]
ITSR	5.8S-ITS2-28S	5'-TCCTCCGCTTATTGATATGC	56.4	Barr et al. [22, 27]
425F	ITS2 forward primer for real-time PCR	5'-ACAAYACCAGAGGGGGTYGC	60.5–64.6	This study
568R	ITS2 reverse primer for real-time PCR	5'-CGTCGATGCGCTCTTCGG	60.8	This study
QP-Harm-ITS2-P8	ITS2 real-time PCR probe for *H*. *armigera*	5'-FAM-TGTCGTCCGYTTTAGCGTGAGAC-BHQ-1	64.6–66.6	This study
RT-ITS-zea	ITS2 real-time PCR probe for *H*. *zea*	5'-HEX-CAACGCCATTAGTAGGCGGACTC-BHQ-1	66.6	This study

### Real-time PCR multiplex protocol

Initial real-time PCR experiments were performed on a Roche LightCycler 480 Real-time PCR System (Roche Diagnostics Corp., Indianapolis, Indiana). Unless stated otherwise, all other assay development and testing was performed on a Bio-Rad CFX96 Touch Real-time PCR Detection System (Bio-Rad Laboratories, Inc., Hercules, Calif.). Development of real-time PCR protocols followed the MIQE Guidelines whenever possible [20]. All real-time PCR reactions used Roche LightCycler 480 Probes Master 2× hot start master mix (Roche Diagnostics Corp.) although master mixes from other vendors were tested in limited quantities (see below). The ITS2 (FAM or HEX) and 18S rDNA (Quasar 670) probe systems were first optimized as duplex assays and then combined into a triplex assay. Assay conditions were optimized so that the 18S rDNA control probe was less sensitive than the diagnostic ITS2 probes in order to limit the probability of false negatives [22, 27] and the control/diagnostic probe combinations generated consistent quantification cycle (Cq) differences of less than seven cycles.

Bio-Rad CFX Manager 3.1 (Bio-Rad Laboratories, Inc.) was used to manage all real-time PCR analyses on the CFX96 Touch instrument. Quantification cycle determination mode was set to “single threshold” and baseline setting was set to “baseline subtracted curve fit.” Baseline cycles and single threshold were set to “auto calculated” for initial testing. However, both diagnostic probes often exhibited a low level of amplification on the alternate species (e.g., the *H*. *armigera* probe with *H*. *zea*) that exceeded the automatically calculated baseline threshold. This resulted in “false positive” Cq values with end relative fluorescence unit (RFU) values that never exceeded 1,000 (usually < 500). Because it would be difficult to replicate standardized samples across different platforms and locations to set a baseline threshold, we changed the threshold setting from “auto calculated” to “user defined” with the value set to “1000.00” for all runs.

The optimized triplex real-time PCR assay was performed in 20 μl reactions consisting of the following (concentrations listed are final): 10 μl 2× master mix; 1 μl each of the forward and reverse ITS2 and 18S rDNA primers (0.5 μM); 0.5 μl each of the *H*. *armigera* and *H*. *zea* probes (0.2 μM); 1 μl of the 18S rDNA control probe (0.4 μM); 3 μl of sterile water; and 1 μl of DNA template. Real-time PCR conditions were as follows: 95°C for 7 min 30 sec (4.4°C/sec ramp rate); 40 cycles of 95°C for 10 sec (4.4°C/sec ramp rate), 62°C for 20 sec (2.2°C/sec ramp rate); and 40°C for 10 sec (1.5°C/sec ramp rate). A plate read was set to occur at the end of each 62°C cycle. All real-time PCR reactions were performed in Roche LightCycler 480 white 96 multi-well plates (Roche Diagnostics Corp.). The triplex real-time PCR assay was tested in two independent runs on all 452 samples.

### Sensitivity analyses

Sensitivity of the real-time PCR assay was evaluated using a series of serial dilutions of both *H*. *armigera* and *H*. *zea* samples. Three samples of each species with known DNA concentrations (as estimated using a NanoDrop 1000) were diluted to a 100 ng/μl stock solution with sterile water and then a 10-fold dilution series was created using the previous solution and sterile water in a 1:9 ratio at each step. Each concentration of the dilution series for each species was tested in triplex (Quasar 670, FAM, HEX), duplex (Quasar 670 and FAM or HEX), and simplex (only FAM or HEX) to observe effects of DNA concentration on the assay and any possible complications created by multiplexing the three probes. Real-time PCR conditions were the same as those listed above with sterile water replacing the unused probe volumes in duplex and simplex reactions. The results from each of the three samples of each species were averaged and the Cq values plotted against DNA concentration on a logarithmic scale. Slopes, y-intercepts, and correlation coefficients were calculated in Excel for the triplex reactions.

### Master mix testing

The effect of different real-time PCR master mix solutions on assay performance was evaluated using kits from three manufacturers. Three 96-well plates with identical samples (94 moth samples + 2 no template controls) were processed on the Bio-Rad CFX96 using the following kits: Roche LightCycler 480 Probes Master 2× hot start master mix (Roche Diagnostics Corp.); Takara Premix Ex Taq (Perfect Real Time) DNA Polymerase (Clontech Laboratories, Inc., Mountain View, Calif.); and Bio-Rad iTaq Universal Probes Supermix (Bio-Rad Laboratories, Inc.).

### Mixed template experiments

Spike experiments were performed to determine the sensitivity of the real-time PCR assay to contamination (e.g., mixed samples). Template DNA (100 ng/μl) from *H*. *armigera* or *H*. *zea* was spiked with DNA of the other species (100 ng/μl starting concentration) across a 10-fold dilution series in ratios of 1:1, 1:10, 1:100, and 1:1000 and tested using the triplex real-time PCR assay. Three replicates of each ratio were tested for each species.

Additional spike experiments were used to determine if the real-time PCR assay is capable of detecting a single *H*. *armigera* among numerous *H*. *zea* while processing bulk trap samples. To replicate potential bulk extractions from trap samples, legs of *H*. *armigera* were combined with legs of *H*. *zea* in ratios (*armigera*:*zea*) of 1:1, 1:4, 1:9, and 1:19 in individual 1.5 ml microcentrifuge tubes. Legs were crushed using a pestle and DNA extracted following the single column protocol listed above. Five replicates of each ratio were extracted and tested using the triplex real-time PCR assay.

### Assay precision testing

Precision, specifically intermediate precision and reproducibility, of the real-time PCR multiplex protocol was evaluated by testing at Mission Lab using a Cepheid SmartCycler II (Cepheid, Sunnyvale, Calif.). All real-time PCR reactions used Takara Premix Ex Taq (Perfect Real Time) DNA Polymerase (Clontech Laboratories, Inc., Mountain View, Calif.). Primer and probe sequences were identical to those in Table 2. Probes were ordered with different fluorophores for better compatibility with the Cepheid system: CAL Fluor Red 610 (Texas Red) for the 18S rDNA control probe, TET for the *H*. *zea* probe, and FAM for the *H*. *armigera* probe. Reactions were performed in triplex with probes and primers at 10 μM working stock (1 μl of each primer, 1 μl of the control probe, and 0.5 μl of each ITS2 probe). Real-time PCR conditions were identical to those already reported (62°C annealing temperature). The Cepheid SmartCycler Software used the default threshold (30 fluorescent units) and growth curve (primary curve) settings. Aliquots of 32 *H*. *armigera* extracts from the Fort Collins lab were used to test the triplex assay. Sensitivity tests using serial dilutions and spike experiments using leg ratios (described above) were also repeated on the Cepheid system.

## Results

### DNA extraction, primer and probe development

DNA concentrations ranged from 3.0 to 878.5 ng/μl (mean 125.4 ng/μl; average of two Nanodrop readings) for the subset of samples measured (approximately 60 *H*. *armigera* and 140 *H*. *zea*). Freshly collected specimens generally had higher DNA concentrations than those obtained from CAPS traps, most of which remained in the field for several weeks.

The 18S rDNA control primers and probe used by Barr et al. [22] produced positive results for all Heliothinae specimens tested. With the exception of replacing CAL Red 610 with Quasar 670 as the fluorophore for the probe, no other sequence modifications to the 18S rDNA markers were made for this study.

**Fig 1:**
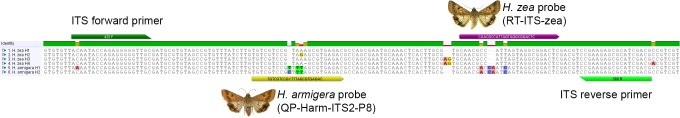
Alignment of ITS2 for *H*. *armigera* and *H*. *zea* showing sequence variation along with primer and probe locations.

Direct sequencing and cloning of PCR products resulted in multiple distinct copies of ITS2 per species along with multiple copies in single individuals. The primers 425F and 568R were selected to maximize differences between *H*. *armigera* and *H*. *zea* while minimizing intraspecific and intra-individual variation. Two *H*. *armigera* and four *H*. *zea* haplotypes were identified within the amplified region of ITS2—these are illustrated in the alignment in Fig 1 along with primer and probe locations.

*Helicoverpa armigera* probe development focused on a 5-bp region that was variable in *H*. *zea* and consistently CTTTA or TTTTA for *H*. *armigera*. Initial 15-bp probes incorporating LNAs targeted at binding to the middle of this region were successful but all also bound to *H*. *zea* with varying levels of efficiently. Moving the target region (and associated LNAs) to the 5’ or 3’ end of the probe reduced binding efficiency for both species. Because non-LNA probes with additional bases added to maintain a similar Tm (total 23–28 bp) produced similar results, subsequent probes were developed without LNAs. The probe “QP-Harm-ITS2-P8” was selected because it produced consistent binding (low Cq values) with *H*. *armigera* and only low levels of binding (high Cq values and low RFUs) with *H*. *zea*. This probe was designed with a “Y” as the 10^th^ base to account for C/T variability in the two *H*. *armigera* haplotypes.

*Helicoverpa zea* probe development focused on a region further 3’ of the *H*. *armigera* probe with a 2-bp indel flanked by two base pair differences from the *H*. *armigera* haplotypes (Fig 1). A 23-bp probe “RT-ITS-zea” was designed that bound consistently to *H*. *zea*.

**Fig 2:**
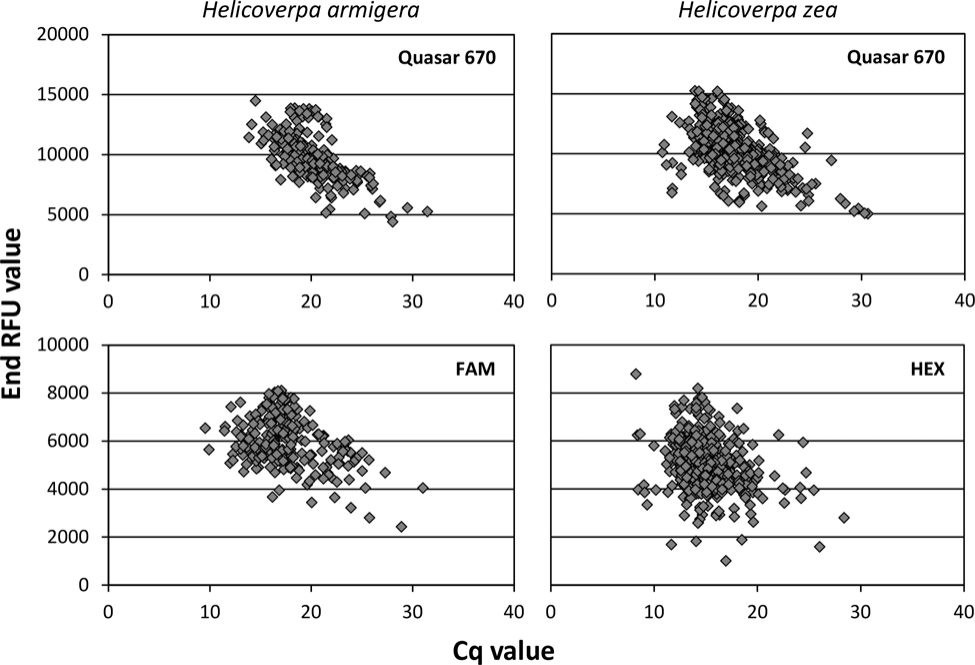
Cq values plotted against end RFU values for each probe in the triplex real-time PCR assay.

All probes exhibited low levels of binding with the alternate species (*H*. *armigera* probe with *H*. *zea* or *H*. *zea* probe with *H*. *armigera*); however, end RFU values in these instances never exceeded 1,000 (usually < 500). Higher annealing temperatures were tested to attempt to reduce incorrect probe binding, but temperatures > 62°C interfered with performance of the 18S rDNA control probe when multiplexed. The problem of positive Cq values with low end RFUs was eliminated by manually adjusting the threshold level to “1000.00” in the CFX Manager software. When binding to the correct species, all three probes returned Cq values with end RFUs in excess of 2,000 for most samples; see Fig 2 for plots of these values.

### Performance of triplex assay on *H*. *armigera*

All 139 of the *H*. *armigera* samples tested with the triplex real-time PCR assay generated Cq values for the control (Quasar 670) and target (FAM) probes. Quasar 670 Cq values ranged from 13.87 to 31.45 (mean 20.15 ± SD 2.80) and FAM Cq values ranged from 9.55 to 31.01 (mean 17.58 ± SD 3.37). Only one *H*. *armigera* specimen (HELICOV-595) had a FAM Cq value > 30 on one run (Cq = 31.01); all other specimens had Cq values ≤ 28.89.

**Fig 3:**
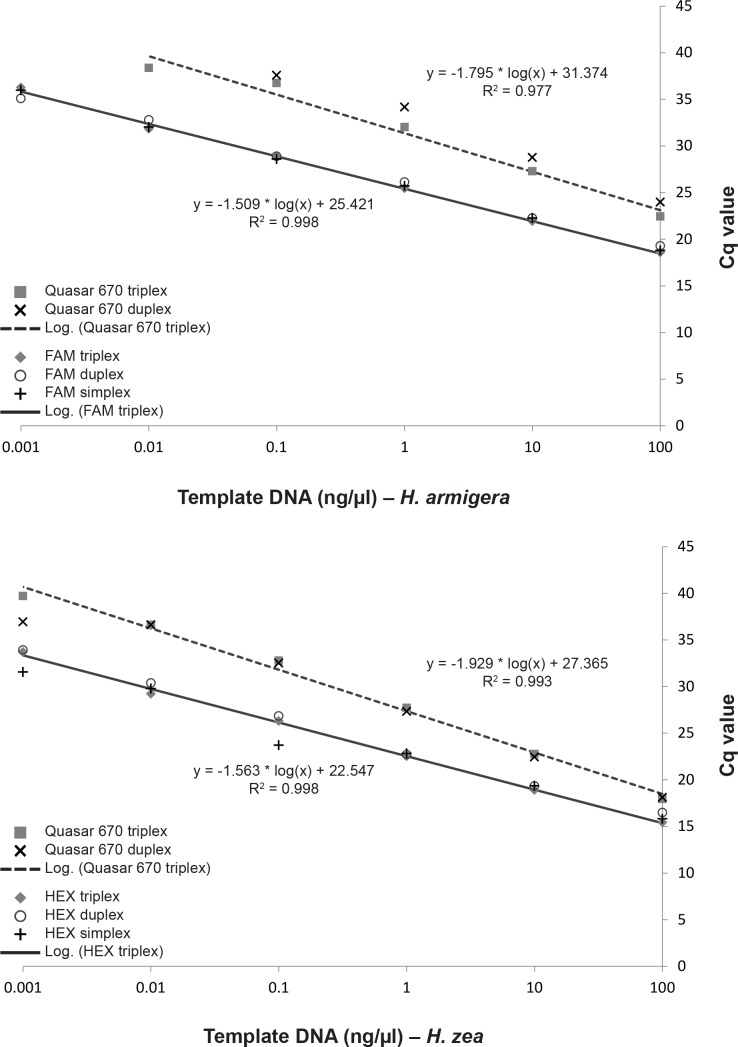
Standard curve of Cq values for serial dilutions of *H*. *armigera* and *H*. *zea* DNA run with the real-time PCR assay in triplex, duplex, and simplex for the ITS2 probe (FAM or HEX) and in triplex and duplex for the control probe (Quasar 670).

As demonstrated by the standard curve of Cq values plotted versus DNA template concentrations (Fig 3), Cq values decrease as DNA template concentration increases in a linear fashion. The FAM probe generated the same Cq values for each DNA concentration regardless if run in simplex, duplex (with Quasar 670), or triplex (with Quasar 670 and HEX). Similarly, the Quasar 670 probe generated the same Cq values for each DNA concentration if run in duplex (with FAM) or triplex (with FAM and HEX). No interactive or negative effects of multiplexing the three probe systems were observed. The difference between Quasar 670 and FAM probe Cq values for an individual is reported as ΔCq (i.e., Quasar 670 Cq − FAM Cq) and used to detect an association between Cq values of the target and control probes. The ΔCq values ranged from 0.09 to 6.68 (mean 3.41 ± SD 1.07). In all cases the control (Quasar 670) was higher than the diagnostic (FAM) probe Cq values.

### Performance of triplex assay on *H*. *zea*

All 258 of the *H*. *zea* samples tested with the triplex real-time PCR assay generated Cq values for the control (Quasar 670) and target (HEX) probes. Quasar 670 Cq values ranged from 10.74 to 30.62 (mean 17.58 ± SD 2.89) and HEX Cq values ranged from 8.24 to 28.40 (mean 14.94 ± SD 2.48). One *H*. *zea* (HELICOV-207) failed for both probes on one of the two runs, while an adjacent sample (HELICOV-208) failed for only the *H*. *zea* probe on one run; it is unknown if these failures were due to pipetting error. Two other samples produced consistently high or low Cq values relative to the other samples. Sample HELICOV-026 produced consistently low Cq values for HEX (8.24–8.65) and consistently high Cq values for Quasar 670 (27.10–30.62), with the Cq difference between the two probes well outside of the mean for all other samples. Sample HELICOV-333 produced consistently high Cq values for HEX (26.02–28.40) and similar but slightly lower values for Quasar 670 (24.86–27.95). Although the identification of both samples was verified using morphology, their molecular identity would remain “inconclusive” using the interpretation rules developed here (see below).

Similar to results for *H*. *armigera*, Cq values decrease as DNA template concentration increases in a linear fashion (Fig 3), and no negative effects of multiplexing the three probe systems were observed. The Quasar 670 probe Cq values were consistently higher than those of the HEX probe for all specimens except the two listed above and two additional specimens (HELICOV-373, 374) that had HEX Cq values exceeding the Quasar 670 Cq values for only the first run; these two samples were within the range of other specimens on the second run. The ΔCq values ranged from 0.04 to 6.16 (mean 2.64 ± SD 0.86) with the Quasar 670 Cq values consistently higher than the diagnostic (HEX) probe Cq values.

### Performance of triplex assay on non-target Heliothinae

All of the 55 non-target Heliothinae samples tested with the triplex real-time PCR assay generated Cq values for the control (Quasar 670) probe ranging from 12.60–39.71 (mean 21.92 ± SD 4.83). None of the non-target Heliothinae samples generated positive Cq values above the threshold level for the *H*. *armigera* or *H*. *zea* probes.

### Master mix testing

Results of the master mix testing are listed in Table 3. The Roche LightCycler 480 Probes Master performed more consistently (lowest SD) across all three probes. A one-way ANOVA revealed statistical significance between kits for the control (Quasar 670) probe (P = 0.015) and *H*. *armigera* (FAM) probe (P = 0.041). No significant difference was found for the *H*. *zea* (HEX) probe (P = 0.196). None of the kits produced Cq values outside the range of those specified in the interpretation rules, and none of the samples would have been scored differently using different master mixes.

**Table 3:** Cq values for each probe resulting from testing different real-time PCR master mixes with the triplex assay. ANOVA statistics for comparisons of each data set are provided in the last row of the table.

	Quasar 670 (Control)—Cq values				FAM (*H*. *armigera*)—Cq values				HEX (*H*. *zea*)—Cq values			
	Min.	Max.	Mean	± SD	Min.	Max.	Mean	± SD	Min.	Max.	Mean	± SD
Roche LightCycler 480 Probes Master	14.11	18.44	15.77	0.92	14.22	23.06	16.99	1.45	11.81	15.75	13.26	0.81
Takara Premix Ex Taq (Perfect Real Time)	12.44	23.94	15.32	2.71	13.20	23.62	16.31	1.85	10.54	20.27	13.02	2.28
Bio-Rad iTaq Universal Probes Supermix	13.80	23.68	16.48	2.55	12.88	22.17	15.95	1.61	11.16	19.64	13.55	2.21
	Statistics:	*df* = 2	*F ratio* = 4.25	*P* = 0.015	Statistics:	*df* = 2	*F ratio* = 3.29	*P* = 0.041	Statistics:	*df* = 2	*F ratio* = 1.64	*P* = 0.196

### Mixed template experiments

When *H*. *armigera* DNA was spiked with *H*. *zea* DNA in ratios (*armigera*:*zea*) of 1:1, 1:10, 1:100, and 1:1000, and tested with the triplex real-time PCR assay, only the first ratio (1:1) produced Cq values above the threshold level for the *H*. *armigera* (FAM) probe (mean Cq = 19.35). When *H*. *zea* DNA was spiked with *H*. *armigera* DNA in ratios (*zea*:*armigera*) of 1:1, 1:10, 1:100, and 1:1000, the *H*. *zea* (HEX) probe returned Cq values for the ratios 1:1 (mean Cq = 16.70), 1:10 (mean Cq = 19.85), and 1:100 (mean Cq = 22.77).

Bulk leg extractions produced similar results for the *H*. *armigera* (FAM) probe. Only the 1:1 ratio extraction (1 leg of *H*. *armigera* + 1 leg of *H*. *zea*) returned Cq values (mean Cq = 21.02). All other ratios (1:4, 1:9, and 1:19) did not produce Cq values above the threshold level.

### Assay precision

All 32 of the *H*. *armigera* samples tested with the triplex real-time PCR assay on the Cepheid system generated Cq values for the control (Texas Red) probe that ranged from 18.21 to 24.17 (mean 20.40 ± SD 1.38). All but one of the samples generated a Cq > 0 for the *H*. *armigera* (FAM) probe, with values ranging from 16.71–20.79 (mean 18.58 ± SD 0.99). This sample (HELICOV-107) generated a similar result after repeating the experiment on all 32 samples. However, analysis of an independent DNA extraction of that same sample (larva) generated a Cq for the *H*. *armigera* probe. It is not clear if the initial failure was due to degradation of nucleic acids in the first extraction sample, reduced sensitivity of the Cepheid protocol, and/or another factor. Consequently, this sample is recorded as a failure in the intermediate precision assay (i.e., how the assay performs on a different platform using identical samples). None of the samples produced a Cq value for the *H*. *zea* (TET) probe. The control (Texas Red) Cq values were higher than those of the target (FAM) for all samples (mean ΔCq = 1.78 ± SD 0.65).

Based on serial dilution data, the Cepheid system is less sensitive than the Bio-Rad CFX96. Samples of *H*. *armigera* failed to register a Cq > 0 for DNA concentrations less than 1.0 ng/μl for the *H*. *armigera* (FAM) probe. Samples of *H*. *zea* produced Cq values > 0 for concentrations down to 0. 1 ng/μl for the *H*. *zea* (TET) probe; however, the control probe (Texas Red) failed to produce Cq values > 0 below DNA concentrations of 1.0 ng/μl for these same samples. The control (Texas Red) Cq values were higher than those of the targets (FAM, TET) for all samples with DNA concentrations ≥ 1.0 ng/μl (mean ΔCq = 3.68 ± SD 1.44).

Aliquots of the bulk leg extractions produced results similar to those of the Bio-Rad CFX96. Only the 1:1 ratio extraction (1 leg of *H*. *armigera* + 1 leg of *H*. *zea*) returned Cq values > 0 (mean Cq = 20.47 ± SD 0.45). All other ratios (1:4, 1:9, and 1:19) did not produce Cq values > 0.

### Development of interpretation rules

The interpretation rules developed here are based on the Cq values obtained from the 452 samples tested with the real-time PCR assay on a Bio-Rad CFX96 Touch Real-time PCR Detection System. As demonstrated by testing on a Cepheid SmartCycler II, modification of the rules presented here may be desirable if the assay is performed on other real-time PCR systems. Simply detecting fluorescence in a reaction (e.g., DNA is present) is not sufficient because the source of the DNA is not always known [22]. Abnormally low or high Cq values could be the result of contamination, equipment malfunction, incorrect assay preparation, non-specific probe binding, or low quality DNA extract. The rules presented here are designed to function with target DNA concentrations as low as 0.01 ng/μl.

**Fig 4:**
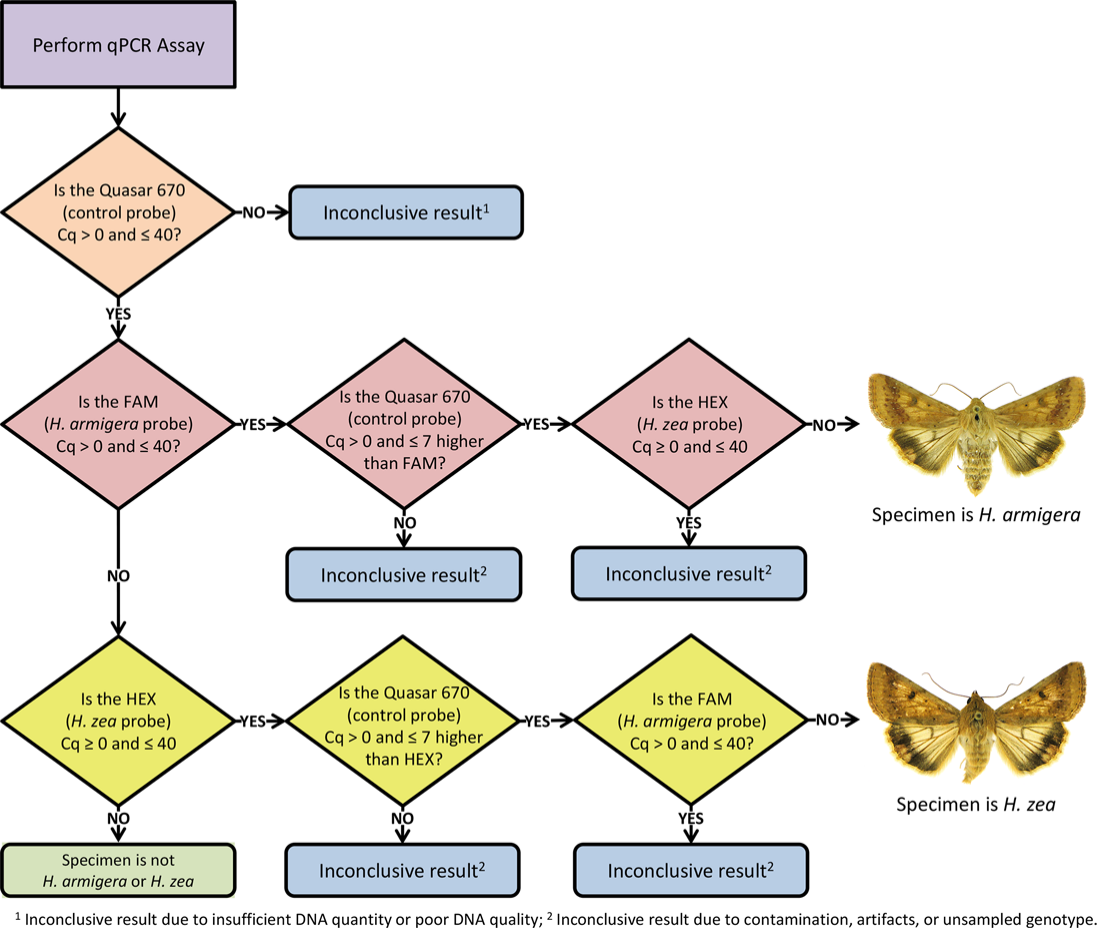
Decision tree flowchart developed using the interpretation rules for the triplex real-time PCR assay run on a Bio-Rad CFX96 system.

The following interpretation rules were developed: 1) the control (Quasar 670) probe Cq value must be > 0 and ≤ 40 to ensure DNA is present in concentrations sufficient for analysis; 2) the target (FAM or HEX) probe Cq value must be > 0 and ≤ 40; 3) the difference between the control (Quasar 670) probe and target (FAM or HEX) probe Cq values must be > 0 and ≤ 7 (the control Cq value must be higher); and 4) both target probes (FAM and HEX) cannot have Cq values > 0 and ≤ 40 at the same time. These interpretation rules are the basis for the decision tree illustrated in Fig 4.

## Discussion

We have developed a real-time PCR assay using the internal transcribed spacer region 2 (ITS2) locus as a diagnostic marker and the 18S rDNA locus as an internal control that is capable of diagnosing *H*. *armigera* and *H*. *zea* larvae and adults intercepted at ports of entry and captured during domestic surveys. The assay can be run in triplex with no negative effects on sensitivity. Total analysis time is approximately 50 minutes for isolated DNA. This is a significant time savings over other methods, such as DNA barcoding, where sequencing of the final PCR product is required. The assay is capable of providing a correct diagnosis in the presence of other New World Heliothinae such as *C*. *virescens*.

Four hundred and fifty-two samples were assayed in the study, including 139 *H*. *armigera*, 258 *H*. *zea*, and 55 other Heliothinae. The assay returned the correct diagnosis for 99% of these samples in both runs of the assay. Only four samples generated inconclusive data: one *H*. *armigera* (on one run) generated a high Cq value for the *H*. *armigera* probe; one *H*. *zea* (on one run) generated Cq values of 0 for *H*. *zea* and control probes (likely due to pipetting error); and two *H*. *zea* (on both runs) generated Cq values for *H*.*zea* probe and the control probe (i.e., correct products) but at values outside of the range defined in the interpretation rules for an identification. A single *H*. *zea* (on one run) failed to generate a Cq value for the *H*. *zea* probe—this is the only result that would be considered a false negative. No false negatives were observed for the *H*. *armigera* probe.

No false positives were generated from any samples once the baseline threshold was established. Standard curves demonstrate that the assay is applicable to DNA concentrations of ≥ 0.01 ng/μl. The coefficient of determination (r^2^) for both ITS2 probes was 0.998 (Fig 3); an r^2^ > 0.980 is recommended to maintain amplification efficiency across different starting template copy numbers [32].

Although the assay was tested with a variety of other Helothinae, including other *Helicoverpa*, caution should be used when applying these protocols outside of the New World. We were unable to obtain specimens of two other important Old World *Helicoverpa* pests: *H*. *punctigera* and *H*. *assulta*. *Helicoverpa punctigera* is an important polyphagous agricultural pest in Australia and New Zealand [2]. *Helicoverpa assulta* is an important pest of Solanaceae in East Asia; it also occurs in Africa, Australasia, and the Pacific [2]. We aligned a sequence of ITS2 for *H*. *punctigera* downloaded from Genbank (accession number AF047759.1) [33] with those of *H*. *armigera* and *H*. *zea*, and observed a 12 bp insertion in *H*. *punctigera* that would prevent the *H*. *armigera* (FAM) probe from binding. If this insert is present in all *H*. *punctigera*, the assay will be able to positively separate *H*. *armigera* from *H*. *punctigera*. We were unable to obtain ITS2 sequence data for *H*. *assulta*. Preliminary testing of the triplex real-time PCR assay by Netherlands NPPO on a variety of Heliothinae, including other *Heliothis*, *Protoschinia*, and *Pyrrhia*, resulted in no false positives or false negatives using the interpretation rules developed here (data not shown).

The decision tree in Fig 4 outlines the process used to diagnose a specimen using the interpretation rules developed for the Bio-Rad CFX96 real-time PCR system. The overall process is applicable to any real-time PCR system capable of multiplexing several probes; however baseline threshold levels may need to be adjusted for other systems. We evaluated the intermediate precision of the assay using a Cepheid SmartCycler II in a different laboratory. The Cepheid produced results consistent with the Bio-Rad system, although it was less sensitive, requiring DNA concentrations ≥ 1.0 ng/μl.

Sequencing of ITS2 for *H*. *armigera*, *H*. *zea*, and other Heliothinae revealed multiple distinct copies per species (four in *H*. *zea* and two in *H*. *armigera*) and evidence of intra-individual variation (multiple, distinct copies within a single individual). Direct sequencing of ITS2 was difficult because of intra-individual variability and multiple copies of ITS2 per individual were confirmed in two individuals by cloning PCR products (GenBank accession numbers KT946006–KT946009).

Concerted evolution across rDNA copies is expected to result in intra-genomic uniformity [34], although intra-genomic variation in ITS2 has been documented in several studies [35–36]. We observed similar variability when attempting to sequence ITS2 for Tortricidae [22]. Although intra-individual variation in our moths could be due to the presence of multiple, divergent copies within the genome of each cell (intra-genomic), our data do not distinguish this state from the presence of multiple, divergent copies within the tissue (i.e., cell lineages in the insect have different gene copies [37]). None of the sequences generated from *H*. *zea* specimens were identical to sequences from *H*. *armigera* specimens. We did not attempt to quantify the amount of intra-individual variation within ITS2 for each species.

Mixed template testing demonstrates that the real-time assay developed here is not applicable for diagnosing bulk samples. Detecting a single specimen of *H*. *armigera* in a trap sample predominately composed of *H*. *zea* is desirable for processing pheromone traps. When *H*. *armigera* template DNA was spiked with *H*. *zea* DNA in different ratios and processed as a single sample, the *H*. *armigera* (FAM) probe only produced acceptable Cq values for a 1:1 (100 ng/μl of each) ratio. Based on the standard curve produced for *H*. *armigera*, the assay should be capable of detecting DNA concentrations as low as 0.01 ng/μl. When the experiment was reversed and *H*. *zea* template DNA was spiked with *H*. *armigera* DNA, the assay was capable of detecting *H*. *zea* DNA down to 0.01 ng/μl (1:100 ratio of *zea*:*armigera*), which is towards the lower limit of detection according to the standard curve. Thus, it appears that including *H*. *zea* in the reaction has a negative effect on the *H*. *armigera* (FAM) probe. This could be due to several factors, including excess RNA in *H*. *zea* extractions monopolizing primers, or more copies of ITS2 in *H*. *zea* monopolizing binding of the probes. Preliminary tests of these hypotheses were conducted by increasing the concentration of either ITS2 primers or the FAM probe in real-time PCR reactions. Increasing the concentration of primers up to 8 × had no effect on the results. However, increasing the FAM probe concentration to the same level did produce Cq values > 0 for a small number of samples down to 0.01 ng/μl (1:100 ratio of *armigera*:*zea*). Further testing is required to determine if increasing FAM probe concentration and optimization of PCR conditions can be used to improve results with mixed DNA templates. More problematic for bulk sample processing is the DNA extraction itself. When legs were extracted from individuals of *H*. *armigera* and *H*. *zea* in the same reaction in ratios above 1:1, the assay failed to produce Cq values above the threshold level. Alternate extraction methods may also be necessary to improve results with bulk samples.

Detection of hybrid individuals is also potentially problematic. It has been estimated that *H*. *zea* split from a common ancestor with *H*. *armigera* by a founder event approximately 1.5 million years ago [15]. This hypothesis is supported by DNA sequence data as well as very similar morphology and mating compatibility between the two species. Under laboratory conditions, *H*. *armigera* and *H*. *zea* have been demonstrated to be able to mate and produce fertile offspring [1, 6, 38, 39–40]. We were able to obtain several *armigera* × *zea* hybrids produced during sterility studies performed in Mississippi in the mid-1980s; however DNA extraction for these individuals failed. Thus, we have not tested the real-time assay on any hybrid individuals and cannot hypothesize on how it would function on hybrids. Although no *armigera* × *zea* hybrids have yet to be identified in the wild, recognition of hybrids is potentially problematic for any identification method [4].

Other rapid molecular methods that do not require sequencing have proven successful in separating *H*. *armigera* from other species of *Helicoverpa* present in the Old World [41–42]. Behere et al. [18] developed a PCR-RFLP assay using two regions of mtDNA, COI, and cytochrome *b* (Cyt *b*), to distinguish between *H*. *armigera*, *H*. *assulta*, *H*. *punctigera*, as well as *H*. *zea*. We tested the Behere et al. [18] assay and found it separated *H*. *armigera* from *H*. *zea*, as reported. However, the method is less useful when additional species present in the New World are included in the assay. The PCR protocol for Cyt *b* does not work well for many of these species precluding diagnosis because failure to amplify is not a diagnostic characteristic (data not shown). More troubling is that we found some species such as *Chloridea virescens* generate RFLP patterns identical to those for *H*. *armigera* (GenBank accession numbers KJ460240–KJ460246). *Chloridea virescens* is a common heliothine that is distributed across much of the New World, and is commonly intercepted at ports of entry in the U.S. and the EU (Netherlands).
